# Dose length product to effective dose coefficients in children

**DOI:** 10.1007/s00247-023-05638-1

**Published:** 2023-03-16

**Authors:** Philip W. Chu, Cameron Kofler, Malini Mahendra, Yifei Wang, Cameron A. Chu, Carly Stewart, Bradley N. Delman, Brian Haas, Choonsik Lee, Wesley E. Bolch, Rebecca Smith-Bindman

**Affiliations:** 1 Department of Epidemiology and Biostatistics, University of California San Francisco, 550 16th Street, Box 0560, San Francisco, CA 94143 USA; 2 Department of Radiology, The University of Chicago, Chicago, IL USA; 3 Department of Pediatrics, Division of Pediatric Critical Care, UCSF Benioff Children’s Hospital, University of California San Francisco, San Francisco, CA USA; 4 Philip R. Lee Institute for Health Policy Studies, University of California San Francisco, San Francisco, CA USA; 5 Department of Diagnostic, Molecular and Interventional Radiology, Icahn School of Medicine at Mount Sinai, New York, NY USA; 6 Department of Radiology and Biomedical Imaging, University of California San Francisco, San Francisco, CA USA; 7 Radiation Epidemiology Branch, National Cancer Institute, National Institutes of Health, Bethesda, MD USA; 8 Department of Biomedical Engineering, University of Florida, Gainesville, FL USA; 9 Department of Obstetrics, Gynecology and Reproductive Sciences, University of California San Francisco, San Francisco, CA USA

**Keywords:** Computed tomography, Effective dose coefficients, Registry

## Abstract

**Background:**

The most accurate method for estimating effective dose (the most widely understood metric for tracking patient radiation exposure) from computed tomography (CT) requires time-intensive Monte Carlo simulation. A simpler method multiplies a scalar coefficient by the widely available scanner-reported dose length product (DLP) to estimate effective dose.

**Objective:**

Develop pediatric effective dose coefficients and assess their agreement with Monte Carlo simulation.

**Materials and methods:**

Multicenter, population-based sample of 128,397 pediatric diagnostic CT scans prospectively assembled in 2015–2020 from the University of California San Francisco International CT Dose Registry and the University of Florida library of highly realistic hybrid computational phantoms. We generated effective dose coefficients for seven body regions, stratified by patient age, diameter, and scanner manufacturer. We applied the new coefficients to DLPs to calculate effective doses and assessed their correlations with Monte Carlo radiation transport-generated effective doses.

**Results:**

The reported effective dose coefficients, generally higher than previous studies, varied by body region and decreased in magnitude with increasing age. Coefficients were approximately 4 to 13-fold higher (across body regions) for patients  <1 year old compared with patients 15–21 years old. For example, head CT (54% of scans) dose coefficients decreased from 0.039 to 0.003 mSv/mGy-cm in patients  <1 year old vs. 15–21 years old. There were minimal differences by manufacturer. Using age-based conversion coefficients to estimate effective dose produced moderate to strong correlations with Monte Carlo results (Pearson correlations 0.52–0.80 across body regions).

**Conclusions:**

New pediatric effective dose coefficients update existing literature and can be used to easily estimate effective dose using scanner-reported DLP.

**Supplementary Information:**

The online version contains supplementary material available at 10.1007/s00247-023-05638-1.

## Introduction

The number of CT scans performed in the USA has grown significantly in the last three decades [1], raising population exposure to ionizing radiation from medical imaging [2], yet clinicians ordering CT scans for pediatric patients often underappreciate the radiation and cancer risks caused by these exposures [3]. While the Food and Drug Administration (and campaigns such as Image Gently and Image Wisely) call for improving education and monitoring radiation doses [4], quantifying exposure has been challenging because there are no readily available, easily understood, and widely accepted metrics for quantifying patient exposures and associated cancer risks.

The energy imparted at the time of a CT scan is measured and reported by every modern CT scanner at the time of image acquisition through a dose length product (DLP). DLP reflects the total radiation produced during a scan but cannot quantify radiation absorbed by individual organs, which varies by patient size and age. This is important, as cancer risk depends on the absorbed doses to individual organs; e.g., a child’s future risk of leukemia depends on the doses absorbed by the bone marrow rather than the total machine output. As children grow, the size and locations of organs change; thus, the same DLP delivered to a newborn will be associated with higher organ doses and future cancer risk than the same DLP delivered to an adult, an observation that led to the widespread movement to tailor doses to patient sizes [5].

Effective dose is a measure that combines the radiation generated by the scanner with theoretical modeling of patient age, size, and organ doses to estimate future potential harm (cancer risk). Initially constructed for use in occupational and public health settings and not intended for individual cancer risk prediction [6, 7], it is the only metric that reflects both machine output and patient risk. It is an ideal measure for radiation dose tracking and reporting, as it can be estimated for any radiation source, and the equivalence in units between medical radiation and background radiation makes it simple for clinicians and patients to understand.

The most accurate method of calculating effective dose utilizes Monte Carlo radiation transport techniques to estimate the absorption of photon beams released during a CT scan, as they pass through simulated body tissues of a virtual anthropomorphic phantom [8, 9]. The estimated dose delivered to each organ is multiplied by an organ-specific tissue weighting factor that reflects the radiosensitivity of that organ. Those organ-specific products are then summed to generate a whole-body effective dose that is correlated with future cancer risk. Unfortunately, since highly accurate Monte Carlo simulations are time-consuming, they are impractical for routine clinical practice and are usually performed on demand for acute radiation overdoses.

A simpler estimation method multiplies scanner-reported DLP by a scalar DLP-to-effective dose coefficient (in units of millisieverts (mSv) or microsieverts (µSv) per mGy-cm) [10, 11]. This approach scales the scanner reported value to the patient’s age or size and body region imaged. Effective dose coefficients, also known as *k*-factors or conversion coefficients, have been derived previously but were based on a very limited range of patient sizes and scanner types, and were not validated on actual patient scans [12–17]. This study developed age-, size-, and body region-specific pediatric effective dose coefficients using data from the University of California San Francisco (UCSF) International CT Dose Registry (“Registry”) and a large library of anatomically accurate computational phantoms that permits dose estimation across a broad range of patient characteristics. The objective of this study is to develop new pediatric effective dose coefficients and assess their agreement with more complex Monte Carlo simulation.

## Materials and methods

The study population consisted of all diagnostic CT scans performed between January 1, 2015, and November 02, 2020, in patients aged 0–17 years old in the Registry, and a 5% sampling of patients age 18–21 (to ensure they did not dominate the results). Scans were performed at 151 imaging facilities from 26 healthcare organizations in 20 US states and 7 countries, all of whom used Radimetrics (Bayer HealthCare, Whippany, NJ) dose management software [18–20]. Most pediatric scans (92%) are from US facilities. Perfusion scans, CT exams with missing or erroneous data, or values in the bottom or top one percent for CTDI-vol, DLP, patient diameter, scan-length, effective dose, and mAs were excluded (to remove potential outliers).

For each CT scan performed, Digital Imaging and Communications in Medicine (DICOM) metadata, including patient data (age, sex, average effective diameter), anatomic area (previously validated) [21], scanner (manufacturer and model), and technical parameters (scan length, DLP, kVp, mAs, 16- or 32-cm phantom) were extracted from the Registry. The sample includes *N*=58 unique scanner models reflecting 348 individual scanners from the four largest manufacturers: General Electric (GE) Healthcare, *N*=24 models (Chicago, IL, USA); Siemens, *N*=19 models (Siemens Healthcare, Erlangen, Germany); Philips, *N*=10 models (Koninklijke Philips N.V., Amsterdam, The Netherlands); and Canon Medical Systems Corporation/Toshiba, *N*=5 models (Ōtawara, Tochigi, Japan). The UCSF Committee on Human Research provided a waiver of individual informed consent. Collaborating institutions obtained local Institutional Review Board approval or relied on the UCSF approval to contribute data to the Registry.

### Phantom library

The University of Florida (UF) Department of Radiology, in collaboration with the National Cancer Institute (NCI), created an expansive library of hybrid computational human phantoms [22], including 12 reference size computational phantoms (6 males and 6 females of differing ages) and 351 non-reference computational phantoms. The smallest male and female phantoms are 85 cm in length and 10 kg in weight. The largest phantoms are 185 cm and 125 kg for males and 175 cm and 115 kg for females. As such, the library represents a wide distribution of patient size [22–25].

### Monte Carlo-estimated effective dose

Using patient sex, age, mean effective diameter (mean of individual acquisition effective diameter values) [21], and scan length, all scans were mapped to the closest body morphometry-matched UF/NCI hybrid computational phantom, and technical parameters from each scan (for abdomen and pelvis, chest, combined chest abdomen and pelvis, and spine) were used to simulate scans and calculate organ doses for each scan. Inputs for the Monte Carlo simulations included CT scanner manufacturer, X-ray tube current (mAs), tube potential (kV), scan length, and whether the scan was performed using a fixed mA or under tube current modulation. Head and neck scans did not use diameter for phantom matching. Monte Carlo simulations were performed using previously described methodology to estimate organ doses [23]. The organ doses were multiplied by the ICRP Publication 103 tissue weighting factors and then summed to compute the patient-specific effective dose for each scan.

There was consistent use of 16-cm and 32-cm reporting phantoms (unrelated to computational phantoms) across body regions and age groups: the 16-cm phantom was used for 99% of head scans, and the 32-cm phantom for nearly 100% of neck, cardiac, and spine scans, and for a large majority (>90%) of other scans (Supplementary Table 1). Thus, dose coefficients were not generated separately by phantom size.

### Effective dose coefficient generation

Stratum-specific effective dose coefficients were calculated, defined as the ratio of the scan-specific effective dose (generated using Monte Carlo simulations) to the DLP by body region, age, patient diameter, and manufacturer. Median (and 25th and 75th percentile) coefficients were calculated (in units of microsieverts (mSv) per mGy-cm) for abdomen and pelvis (separate or combined), cardiac, chest, combined chest abdomen and pelvis, head, neck, and thoracic or lumbar spine CT. For simplicity, combined chest abdomen and pelvis includes whole spine exams, head includes sinus and face exams, and neck includes cervical spine exams. The relative coefficients comparing the youngest to oldest age groups, and the smallest to largest diameter groups, were calculated by body region. Analyses were limited to strata with at least 10 scans.

### DLP-derived effective dose based on age and size

For each CT scan in the Registry, the DLP was multiplied by the appropriate age, size, or manufacturer-matched dose coefficient to estimate the DLP-derived effective dose. This was repeated using combined age and size strata-specific dose coefficients.

### Agreement between different approaches for reporting effective dose

Pearson correlation coefficients were used to assess agreement between the Monte Carlo-generated effective doses and the two new DLP-derived effective doses based on age and diameter, respectively. The percentage of scans where the DLP-derived effective dose differed from the Monte Carlo-generated effective dose by 50% or greater was calculated for the two approaches. To compare the newly reported dose coefficients with prior work, we applied the coefficients from Shrimpton [14] to our sample to calculate effective dose and compare those values with our Monte Carlo-derived estimates. We applied Shrimpton’s single-year coefficients to our age groups: “0 year old”=“0 year”; “1 year old”=“1–4 years”; “5 years old”=“5–9 years”; “10 years old”=“10–14 years”; and “adult”=“15–21 years.

Analyses used SAS version 9.3 (SAS Institute, Cary, NC) and R (version 4.1.1 (2021-08-10)).

## Results

A total of 128,397 CT scans in pediatric patients were assembled from the Registry (Table 1). The most common body region was the head (*n*=68,831, 53.6% of scans.) The number of scans increased with increasing age for all body regions except cardiac, even with deliberate under-sampling of the 18–21-year-old group.

**Table 1:** Computed tomography scans included by body region, sex, age, and manufacturer

	Total	Sex				Age (years)					Manufacturer			
		Female		Male		<1	1–4	5–9	10–14	15–21	General Electric	Siemens	Philips	Canon
Body region	*N*	*N*	%	*N*	%	*N*	*N*	*N*	*N*	*N*	*N*	*N*	*N*	*N*
Head	68,831	29,540	50.9	39,291	55.8	5,507	12,428	13,630	17,779	19,487	27,071	31,038	3,964	6,758
Neck	11,131	4,851	8.4	6,280	8.9	158	1,364	1,976	3,202	4,431	3,664	5,935	608	924
Chest	11,253	5,146	8.9	6,107	8.7	864	1,746	1,919	2,773	3,951	4,294	6,411	169	379
Cardiac	524	199	0.3	325	0.5	256	120	49	52	47	59	456	4	5
Abdomen and pelvis	29,512	15,153	26.1	14,359	20.4	345	2,241	5,440	8,738	12,748	10,590	13,913	1,729	3,280
Combined chest abdomen and pelvis	4,229	1,820	3.1	2,409	3.4	80	599	743	1,041	1,766	2,592	1,498	56	83
Thoracic or lumbar spine	2,917	1,316	2.3	1,601	2.3	3	48	213	928	1,725	1,170	1,282	350	115
Total	128,397	58,025	100	70,372	100	7,213	18,546	23,970	34,513	44,155	49,440	60,533	6,880	11,544

### Effective dose coefficients by patient factors

**Fig. 1:**
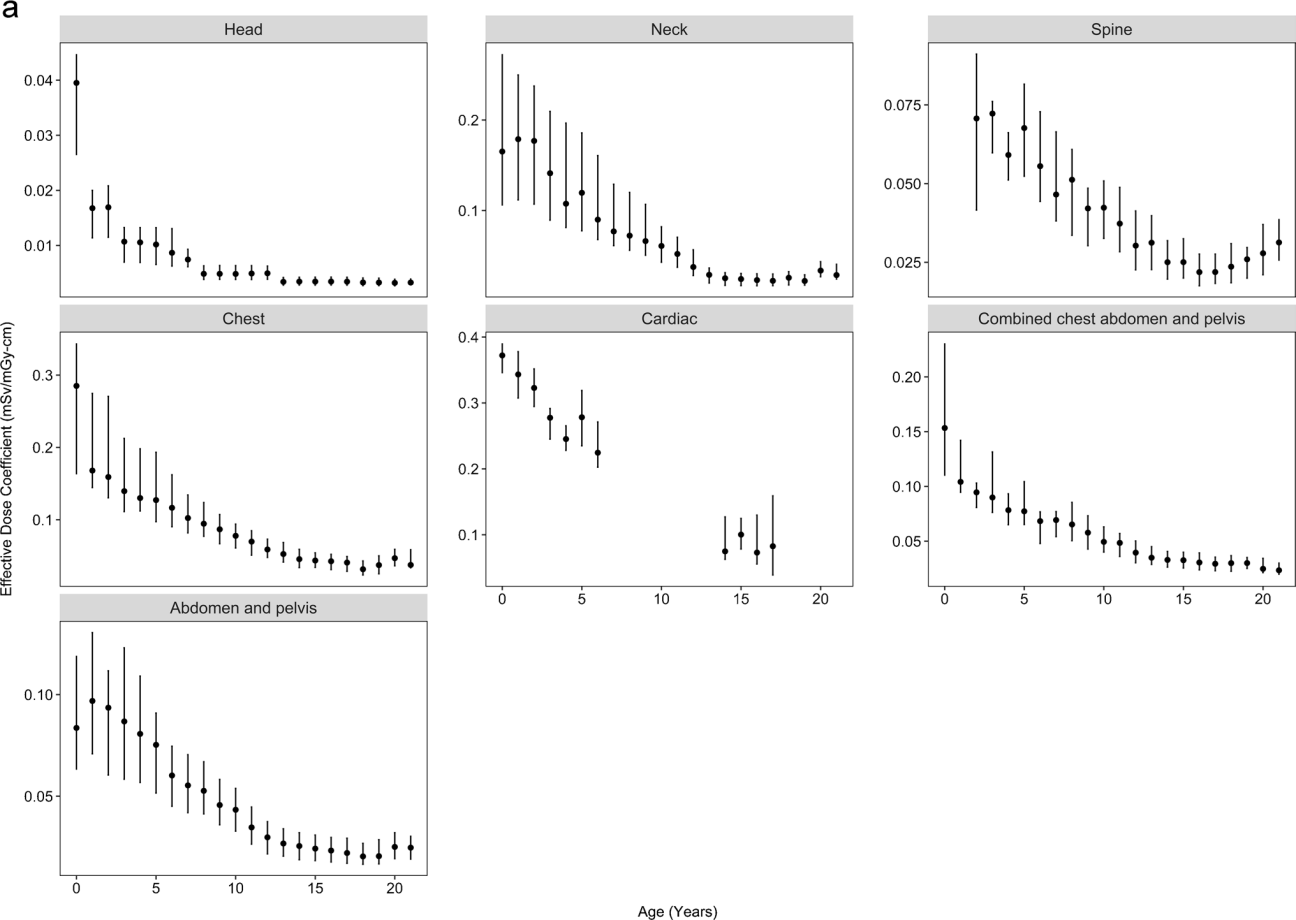

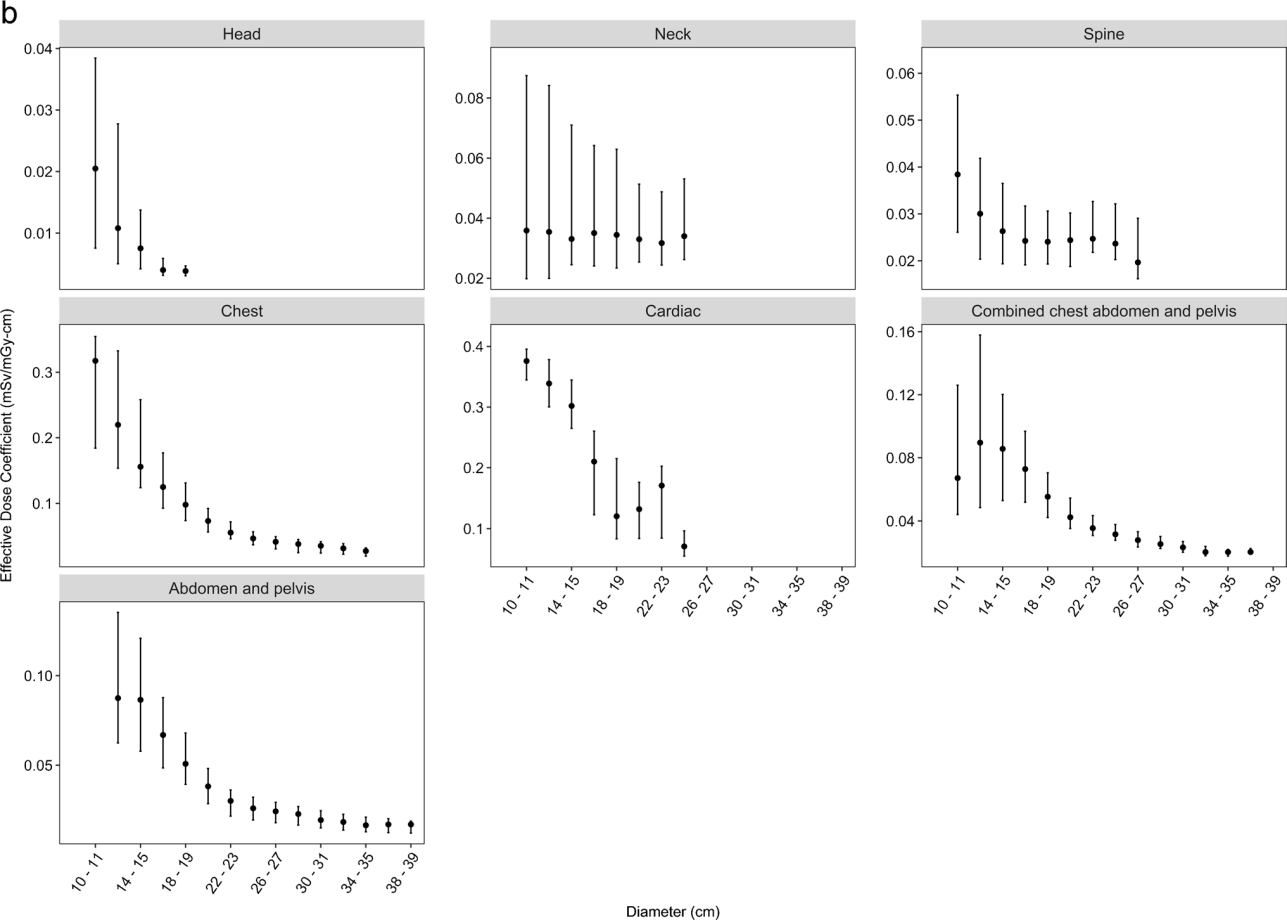
**a** Effective dose coefficients (EDC, in mSv/mGy-cm) by body region and patient age. Solid dots indicate medians. Vertical lines indicate EDC interquartile range (IQR). **b** Effective dose coefficients (in mSv/mGy-cm) by body region and patient diameter. Solid dots indicate medians. Vertical lines indicate EDC interquartile range (IQR)

**Fig. 2:**
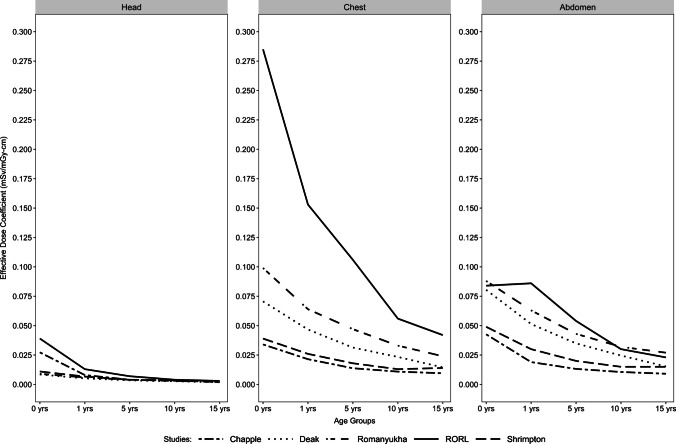
Comparison of effective dose coefficients (in mSv/mGy-cm) from current report (RORL) to prior publications: Chapple et al. 2002 [15], Deak et al. 2010 [16], Romanyukha et al. 2016 [17], Shrimpton 2004 [14]

Median effective dose coefficients declined with increasing age across all body regions (Table 2 and Fig. 1a). The coefficients were approximately 4–13-fold higher for patients under 1 year compared with 15–21 years old across the different body regions. For example, the effective dose coefficient for head CT declined from 0.039 mSv/mGy-cm among patients  <1 year to 0.003 mSv/mGy-cm among patients ages 15–21 (relative coefficient 13.0), and for chest CT declined from 0.285 mSv/mGy-cm among children  <1 year to 0.042 mSv/mGy-cm among patients ages 15–21 (relative coefficient 6.8, Table 2). For most body regions, the steep declines in effective dose coefficients in younger cohorts level off at age 10 years (Fig. 1a). Figure 2 shows the dose coefficients from this study (labeled as “RORL” for “Radiology Outcomes Research Laboratory”) alongside those from previous studies [14–17]. The effective dose coefficients were highest for cardiac and chest CT and lowest for head CT (Table 2 and Fig. 1a).

**Table 2:** Median (25th and 75th percentiles) effective dose coefficients (in mSv/mGy-cm) and relative coefficients comparing youngest to oldest age groups, by body region and patient age

	Age (years)					
Body Region	<1	1–4	5–9	10–14	15–21	Relative coefficient <1 year vs 15–21 years
Head	0.039 (0.026, 0.045)	0.013 (0.009, 0.018)	0.007 (0.005, 0.010)	0.004 (0.003, 0.006)	0.003 (0.003, 0.004)	13.0
Neck	0.165 (0.105, 0.273)	0.143 (0.096, 0.219)	0.080 (0.061, 0.141)	0.034 (0.024, 0.056)	0.024 (0.017, 0.031)	6.9
Chest	0.285 (0.163, 0.344)	0.153 (0.122, 0.232)	0.106 (0.080, 0.144)	0.056 (0.043, 0.074)	0.042 (0.030, 0.052)	6.8
Cardiac	0.372 (0.345, 0.391)	0.302 (0.266, 0.345)	0.217 (0.190, 0.278)	0.097 (0.073, 0.175)	0.094 (0.058, 0.135)	4.0
Abdomen and pelvis	0.084 (0.063, 0.119)	0.086 (0.060, 0.115)	0.054 (0.042, 0.072)	0.030 (0.022, 0.039)	0.023 (0.017, 0.030)	3.7
Combined chest abdomen and pelvis	0.153 (0.110, 0.231)	0.092 (0.073, 0.121)	0.068 (0.051, 0.083)	0.037 (0.030, 0.050)	0.030 (0.023, 0.037)	5.1
Thoracic or lumbar spine	NA	0.063 (0.055, 0.079)	0.048 (0.035, 0.065)	0.030 (0.022, 0.041)	0.023 (0.018, 0.030)	2.7

The effective dose coefficients similarly declined with patient diameter (Table 3 and Fig. 1b). For example, the effective dose coefficient for chest CT declined from 0.171 mSv/mGy-cm among patients with diameters of 11–15 cm to 0.032 mSv/mGy-cm among patients with diameters of 31–35 cm (relative coefficient 5.3).

**Table 3:** Median (25th and 75th percentiles) effective dose coefficients (in mSv/mGy-cm) and relative coefficients comparing smallest to largest diameter groups, by body region and patient size. Head and neck conversion coefficients were not calculated by patient size

Body region	11–15 cm	16–20 cm	21–25 cm	26–30 cm	31–35 cm	36–40 cm	Relative coefficient smallest vs largest diameter
Chest	0.171 (0.132, 0.297)	0.106 (0.076, 0.146)	0.052 (0.043, 0.070)	0.040 (0.027, 0.047)	0.032 (0.022, 0.039)		5.3
Cardiac	0.338 (0.291, 0.373)	0.165 (0.100, 0.238)	0.086 (0.070, 0.143)	0.061 (0.053, 0.072)			5.5
Abdomen and pelvis	0.087 (0.058, 0.124)	0.053 (0.040, 0.074)	0.029 (0.021, 0.035)	0.023 (0.017, 0.028)	0.018 (0.014, 0.023)	0.017 (0.012, 0.020)	5.1
Combined chest abdomen and pelvis	0.085 (0.051, 0.130)	0.063 (0.044, 0.080)	0.035 (0.029, 0.043)	0.026 (0.023, 0.031)	0.021 (0.018, 0.025)	0.020 (0.019, 0.022)	4.3
Thoracic or lumbar spine	0.028 (0.020, 0.040)	0.024 (0.019, 0.031)	0.025 (0.020, 0.032)	0.021 (0.017, 0.029)			1.3

The magnitudes of the relative effective dose coefficients were generally similar for chest, abdomen and pelvis, and combined chest, abdomen, and pelvis whether based on size or age. For example, the median relative dose coefficient for smallest compared with the largest children for chest CT was 5.3 based vs. 6.8 between youngest and oldest children.

### Effective dose coefficients by manufacturer

The effective dose coefficients differed very little by scanner manufacturer (Supplementary Fig. 1). No manufacturer consistently produced the highest or lowest dose coefficients across all body region and age group strata.

### Accuracy of DLP-derived effective dose estimates

For all body regions, broad (5-year) age group DLP-derived effective dose estimates were moderately to strongly correlated with the Monte Carlo-generated effective dose estimates (correlation range 0.52 to 0.80, Table 4). Using single-year age categories elevated the correlations modestly. Diameter-specific DLP-derived effective dose estimates were also moderately to strongly correlated with Monte Carlo-generated effective dose estimates (range 0.60 to 0.80, Table 4). There is no major systematic under- or overestimation of effective dose using the dose coefficients (Supplementary Fig. 2). Using combined age and diameter categories (detailed conversion coefficients provided in Supplementary Table 2) produced only slight improvements in correlations, except for cardiac CT, where the correlations increased from 0.70 (for age) and 0.80 (for diameter) to 0.94 when conversion coefficients simultaneously included age and diameter (Table 4), though we note that cardiac scans are far less common than other body regions in the registry, and this is reflected in the far wider confidence intervals.

**Table 4:** Pearson correlation coefficient between Monte Carlo-generated and DLP-derived effective dose, and proportion of scans whose DLP-derived effective dose differs from the Monte Carlo-generated dose by more than 50%, using body region plus patient age and diameter category-specific effective dose coefficients. Head and neck values for patient diameter are not calculated because Monte Carlo-generated effective dose uses age only

	Age				Diameter		Age-diameter	
	5-year Strata		1-year Strata		2 cm Strata		Combined Strata	
	Correlation	Percent with error >50%	Correlation	Percent with Error >50%	Correlation	Percent with error >50%	Correlation	Percent with error >50%
Head	0.77 (0.76,0.77)	22.8%	0.80 (0.79,0.80)	19.6%				
Neck	0.52 (0.50,0.53)	30.9%	0.59 (0.57,0.60)	29.1%				
Chest	0.70 (0.69,0.70)	26.7%	0.70 (0.69,0.71)	25.9%	0.65 (0.64,0.66)	25.8%	0.72 (0.71,0.73)	23.5%
Cardiac	0.70 (0.65,0.74)	12.8%	0.72 (0.69,0.77)	12.8%	0.80 (0.76,0.83)	13.0%	0.94 (0.88,0.99)	6.3%
Abdomen and pelvis	0.63 (0.62,0.63)	22.1%	0.64 (0.63,0.64)	21.3%	0.60 (0.60,0.61)	20.7%	0.64 (0.63,0.65)	18.6%
Combined chest abdomen and pelvis	0.71 (0.70,0.73)	21.3%	0.72 (0.70,0.73)	20.3%	0.70 (0.68,0.71)	21.6%	0.76 (0.74,0.77)	15.7%
Thoracic or lumbar spine	0.80 (0.78,0.81)	17.7%	0.81 (0.80,0.82)	16.9%	0.78 (0.77,0.79)	22.2%	0.82 (0.81,0.83)	16.3%

The percentage of scans where the broad age group-based DLP-derived effective dose differed from the Monte Carlo-derived effective dose by more than 50% ranged from 13% (cardiac) to 31% (neck) (Table 4). For the most common body regions, head and abdomen, the results were 23% and 22%, respectively. Using single-year age categories reduced those percentages minimally. Diameter-based results were similar for all body regions (Table 4). Combined categories produced minimal improvements, with the exception of cardiac scans, where DLP-derived effective dose estimates based on combined age and diameter strata produced a tighter fit with Monte Carlo-derived estimates (Table 4).

The proportion of CT scans with an effective dose estimate that differed by more than 50% from the Monte Carlo estimated effective dose are considerably higher based on the Shrimpton coefficients. For example, the percent of chest scans differing by more than 50% from the Monte Carlo estimated dose was 90.5% using Shrimpton coefficients vs. 26.7% using the new dose coefficients, and the percent of abdomen and pelvis scans differing by more than 50% was 47.2% using Shrimpton coefficients vs. 22.1% using the new dose coefficients.

## Discussion

Multiplying the universally reported DLP by a scalar coefficient offers a simple, rapid method for clinicians to estimate effective dose, which can be included in the medical record and tracked over time. Using novel methods, an exhaustive computational phantom library, and nearly 130,000 CT scans, this study generated new, more granular effective dose coefficients that when applied to a large sample of pediatric CT scans yielded effective dose estimates that correlated moderately or strongly with Monte Carlo modeling. The conversion coefficients highlight the much higher effective doses that one should expect, for all body regions, in the youngest and smallest patients. For patients whose diameters are far from age-based averages, size-based coefficients may be more accurate than age-based coefficients.

There have been important criticisms of the broad use of effective dose in medicine and hesitancy to use it for individual patients. Some detractors stress that it is a theoretical, derived, mathematical construct and not a physical, measurable quantity. By extension, it should apply only to populations and not to individuals [26–29]. However, similar criticisms could be applied to many epidemiological measures, and many experts continue to advocate for the use of effective dose to monitor patients [30, 31], due to its utility as a predictive value relative to other measurements.

It is important that dose reports delivered to patients clarify that DLP-derived effective dose estimates offer an approximation to patient-specific dose rather than an exact, precise measurement, but still provide the best currently available estimate for routine clinical use. This report demonstrates that refining conversion coefficients results in estimates that are strongly correlated with these more accurate measures. While noting the uncertainties inherent in these calculations, the ICRP concluded that effective dose may be considered an approximate indicator of possible (future cancer) risk [11].

The method of generating effective dose coefficients described herein requires body size-dependent, realistic computational human phantoms. The best estimates would derive from personalized “ideal” phantoms that are anatomically identical to each patient being scanned (reflecting the patient-specific approach favored by the ICRP). While theoretically possible, this is prohibitively time-consuming for routine clinical work. The next best available option, used herein, is a computational phantom designed to be anatomically accurate but not made from the particular CT scan for which the dose calculations are being performed. This study employed the largest library of human computational phantoms available for medical dosimetry and applied these to actual patient scans [27], allowing more accurate mapping of each patient to a phantom matching his/her individual anatomy and body habitus than any previous study.

The effective dose coefficients derived from this study are generally higher than prior studies (Fig. 2), for several reasons. First, nearly all previous studies relied on stylized geometric phantoms (essentially mathematical surface equations) to generate effective dose coefficients [16–21], rather than actual patient data. The geometric shapes used to model human organs in earlier studies were novel when developed but poorly approximate individual organ contours and visceral fat distributions in comparison to the computational phantoms employed in this work. Second, since the UF/NCI library is larger than libraries employed in previous studies, it can more accurately represent a broader range of patient sizes. Third, no previous study tested derived coefficients on actual pediatric patient data. Fourth, most studies used a single scanner or very small number of scanners, contrasting with the large number of scanners included in this report. Fifth, older studies (e.g., [14, 15]) used ICRP-60 tissue weighting factors, which were subsequently updated [10]. Sixth, all prior studies assumed the use of 16-cm (CTDI-vol) calibration reference phantoms for neck scans, whereas our empirical data show that 32-cm phantoms are routinely used in actual practice. Because of different phantom approaches and libraries, scanner models, tissue weighting factors, and inexact comparability due to different age groups, results differing from earliest studies are not surprising. Likewise, the much higher accuracy of our new coefficients compared with Shrimpton [14] coefficients is expected.

Inter-manufacturer differences in effective dose coefficients were modest, and the minimal gains in accuracy do not justify the complexity of employing manufacturer-specific coefficients.

The coefficients vary by body region, radiosensitivity of the exposed tissues, and the efficiency with which the radiation can penetrate to the exposed organs. The coefficients are lowest in the head (where the skull blocks some of the X-rays, and where the brain is not the highest radiosensitive organ) and are highest in the chest and heart where there is little blocking of X-rays by fat, muscle, or bone and where many very radiosensitive organs are located (including the lungs, breast, and thyroid).

Different radiation dose measures have different applications. CTDI-vol, reflecting the average dose per slice, may be most useful for comparing doses across protocols and for technologist education as this reflects the dose output from the scanner, which is directly under their control. Size-specific dose estimates (SSDE), reflecting the CTDI-vol scaled for patient’s size, may be helpful for comparing several institution’s dose output for abdomen CT, or for comparing an individual CT exam against some benchmark; by eliminating size as a cause of observed differences, remaining differences are due to technique alone. We believe that effective dose, the focus on this report, is valuable for reporting radiation dose for several reasons. First, it reflects both the dose emitted by the scanner as well as consideration of the impact of that dose on future cancer risk, which varies by patient size, age, and the parts of the body irradiated. This information is important to patients and providers alike as it reflects an outcome—cancer risk—that they care about. While the CTDI-vol or SSDE may be the same for an abdomen versus a thigh scan, the effective dose may be far higher for the abdomen scan, and patients may therefore care differently about these scans. Patients report they want more information about radiation dose with respect to future cancer risk, and effective dose provides that information. Additionally, effective dose can be added across different types of medical and non-medical exposures and compared, making it an easier metric to understand for patients and providers who order imaging. It is possible to talk about the effective dose from a CT scan compared with the effective dose of a dental X-ray, compared with the cumulative effective dose of a child who has undergone 15 CTs, where such comparisons are not possible for CTDI-vol and SSDE or other dose metrics limited to CT.

Our paper has limitations. We used mid-scan diameter, not weight or body mass index (BMI), to match patients to computational phantoms of varying body habitus. Among patients of similar diameter, body fat distribution and precise organ shapes and positions vary. In addition, the methods make assumptions about exactly what anatomy was imaged/radiated. Moreover, over-scanning or under-scanning that occur in clinical practice can result in selection of sub-optimal phantoms and thus miscalculation of radiation dose to tissues in adjacent anatomy. These unavoidable inaccuracies should minimally impact the results.

The results reflect performance across a range of hospital types including pediatric and adult hospitals. While the radiation doses may be lower in pediatric hospitals, we do not believe this will bias the conversion coefficients as both the numerator (DLP) and denominator (effective dose) that are used to generate the conversion coefficients would both be equally lower, or equally higher, in pediatric hospitals.

## Conclusion

This analysis of pediatric CT scans from a large, multi-center registry produced new effective dose coefficients, stratified by patient age and size, that are reasonably accurate for quickly estimating pediatric effective dose (from reported dose length product [DLP]).

## Supplementary Information

Below is the link to the electronic supplementary material.

Supplementary Table 1 (DOCX 19.8 KB)

Supplementary Figure 1 (DOCX 68.4 KB)

Supplementary Figure 2 (DOCX 383 KB)

Supplementary Table 2 (DOCX 43.1 KB)

## Abbreviations

BMI: Body mass index
CT: Computed tomography
CTDI-vol: Computed tomography dose index volume
DICOM: Digital Imaging and Communications in Medicine
DLP: Dose length product
EDC: Effective dose coefficient
ICRP: International Commission on Radiation Protection
IQR: Interquartile range
GE: General Electric
mAs: X-ray tube current in milliampere-seconds
mSv: Millisieverts
µSv: Microsieverts
NCI: National Cancer Institute
PET-CT: Positron emission tomography computed tomography
SPECT: Single photon emission computed tomography
UCSF: University of California San Francisco
UF: University of Florida
