# Correction to: Dose length product to effective dose coefficients in children

**DOI:** 10.1007/s00247-023-05756-w

**Published:** 2023-09-02

**Authors:** Philip W. Chu, Cameron Kofler, Malini Mahendra, Yifei Wang, Cameron A. Chu, Carly Stewart, Bradley N. Delman, Brian Haas, Choonsik Lee, Wesley E. Bolch, Rebecca Smith‑Bindman

**Affiliations:** 1 Department of Epidemiology and Biostatistics, University of California San Francisco, 550 16th Street, Box 0560, San Francisco, CA 94143 USA; 2 Department of Radiology, The University of Chicago, Chicago, IL USA; 3 Department of Pediatrics, Division of Pediatric Critical Care, UCSF Benioff Children’s Hospital, University of California San Francisco, San Francisco, CA USA; 4 Philip R. Lee Institute for Health Policy Studies, University of California San Francisco, San Francisco, CA USA; 5 Department of Diagnostic, Molecular and Interventional Radiology, Icahn School of Medicine at Mount Sinai, New York, NY USA; 6 Department of Radiology and Biomedical Imaging, University of California San Francisco, San Francisco, CA USA; 7 Radiation Epidemiology Branch, National Cancer Institute, National Institutes of Health, Bethesda, MD USA; 8 Department of Biomedical Engineering, University of Florida, Gainesville, FL USA; 9 Department of Obstetrics, Gynecology and Reproductive Sciences, University of California San Francisco, San Francisco, CA USA

**Correction to** **:** **Pediatric Radiology (2023) 53:1659–1668**

**https://doi.org/10.1007/s00247-023-05638-1**

The original article contains an error in the supplementary materials. The file originally titled “Supplementary file1” was an incorrect file – this has been removed. The order of the supplementary material has been corrected, and two additional previously missing supplemental files.

The original article has been corrected.

