# Supplementary material for: Dose length product to effective dose coefficients in children
Source: Pediatr Radiol. 2023 Mar 16;53(8):1659–68. doi: 10.1007/s00247-023-05638-1 (PMC10359359; doi:10.1007/s00247-023-05638-1)
Supplement: Supplementary file 1 — Supplementary Table 1 (DOCX 19.8 KB) [file 247_2023_5638_MOESM1_ESM.docx]

Supplementary Table 1. Use of reference phantom (16-cm vs. 32-cm) by body region and age.

|  |  |  | Phantom | |
| --- | --- | --- | --- | --- |
|  |  |  | 16-cm | 32-cm |
| Body Region | Age | N | % | % |
|  |  |  |  |  |
| Head | All Ages | 66,403 | 99 | 1 |
|  | <1 Yrs | 5,327 | 100 | 0 |
|  | 1-4 Yrs | 11,922 | 100 | 0 |
|  | 5-9 Yrs | 13,205 | 99 | 1 |
|  | 10-14 Yrs | 17,158 | 99 | 1 |
|  | 15-21 Yrs | 18,791 | 98 | 2 |
|  |  |  |  |  |
| Neck | All Ages | 10,706 | 0 | 100 |
|  | <1 Yrs | 148 | 0 | 100 |
|  | 1-4 Yrs | 1,317 | 0 | 100 |
|  | 5-9 Yrs | 1,923 | 0 | 100 |
|  | 10-14 Yrs | 3,082 | 1 | 99 |
|  | 15-21 Yrs | 4,236 | 0 | 100 |
|  |  |  |  |  |
| Chest | All Ages | 10,742 | 6 | 94 |
|  | <1 Yrs | 821 | 13 | 87 |
|  | 1-4 Yrs | 1,662 | 15 | 85 |
|  | 5-9 Yrs | 1,832 | 10 | 90 |
|  | 10-14 Yrs | 2,633 | 3 | 97 |
|  | 15-21 Yrs | 3,794 | 1 | 99 |
|  |  |  |  |  |
| Cardiac | All Ages | 518 | 0 | 100 |
|  | <1 Yrs | 256 | 0 | 100 |
|  | 1-4 Yrs | 120 | 0 | 100 |
|  | 5-9 Yrs | 46 | 0 | 100 |
|  | 10-14 Yrs | 50 | 0 | 100 |
|  | 15-21 Yrs | 46 | 0 | 100 |

Supplementary Table 1. continued.

| Abdomen and pelvis | All Ages | 28,324 | 5 | 95 |
| --- | --- | --- | --- | --- |
|  | <1 Yrs | 337 | 28 | 72 |
|  | 1-4 Yrs | 2,162 | 21 | 79 |
|  | 5-9 Yrs | 5,217 | 12 | 88 |
|  | 10-14 Yrs | 8,389 | 3 | 97 |
|  | 15-21 Yrs | 12,219 | 0 | 100 |
|  |  |  |  |  |
| Combined chest abdomen and pelvis | All Ages | 4,031 | 6 | 94 |
|  | <1 Yrs | 80 | 20 | 80 |
|  | 1-4 Yrs | 589 | 21 | 79 |
|  | 5-9 Yrs | 729 | 12 | 88 |
|  | 10-14 Yrs | 1,015 | 1 | 99 |
|  | 15-21 Yrs | 1,618 | 0 | 100 |
|  |  |  |  |  |
| Spine | All Ages | 2,796 | 0 | 100 |
|  | <1 Yrs | 3 | 0 | 100 |
|  | 1-4 Yrs | 46 | 0 | 100 |
|  | 5-9 Yrs | 204 | 0 | 100 |
|  | 10-14 Yrs | 884 | 0 | 100 |
|  | 15-21 Yrs | 1,659 | 0 | 100 |
