# Supplementary material for: Dose length product to effective dose coefficients in children
Source: Pediatr Radiol. 2023 Mar 16;53(8):1659–68. doi: 10.1007/s00247-023-05638-1 (PMC10359359; doi:10.1007/s00247-023-05638-1)

Supplementary Figure 1. Effective dose coefficients (in mSv/mGy-cm) by body region, manufacturer, and patient age.


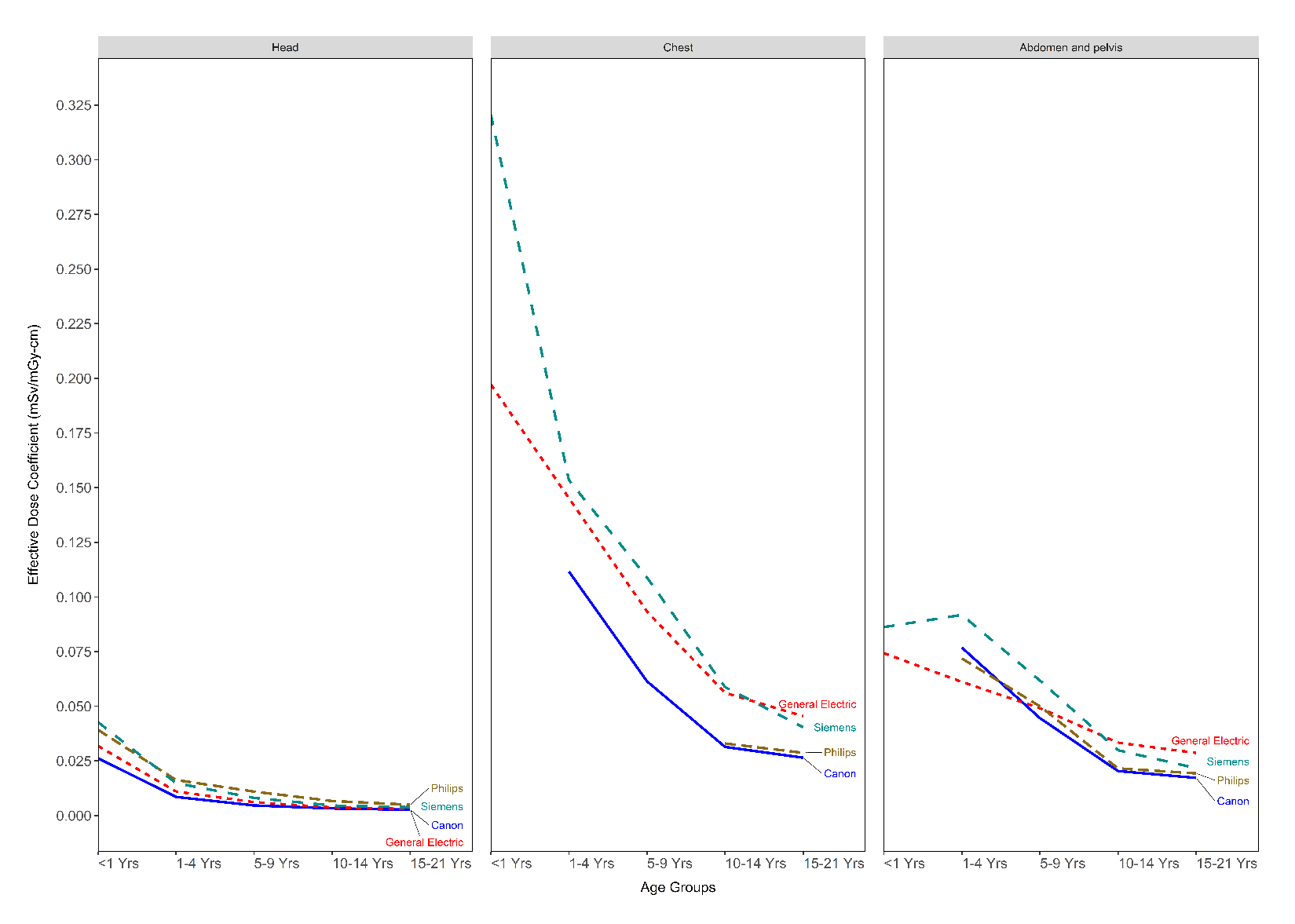

Supplement: Supplementary file 2 — Supplementary Figure 1 (DOCX 68.4 KB) [file 247_2023_5638_MOESM2_ESM.docx]
