# Supplementary material for: Dose length product to effective dose coefficients in children
Source: Pediatr Radiol. 2023 Mar 16;53(8):1659–68. doi: 10.1007/s00247-023-05638-1 (PMC10359359; doi:10.1007/s00247-023-05638-1)
Supplement: Supplementary file 3 — Supplementary Figure 2 (DOCX 383 KB) [file 247_2023_5638_MOESM3_ESM.docx]

Supplementary Figure 2. Comparison of Monte Carlo generated effective dose (ED, in mSv) vs. effective dose generated using the new, diameter-based, effective dose coefficients for abdomen and pelvis scans.
