# Supplementary material for: Dose length product to effective dose coefficients in children
Source: Pediatr Radiol. 2023 Mar 16;53(8):1659–68. doi: 10.1007/s00247-023-05638-1 (PMC10359359; doi:10.1007/s00247-023-05638-1)
Supplement: Supplementary file 4 — Supplementary Table 2 (DOCX 43.1 KB) [file 247_2023_5638_MOESM4_ESM.docx]

Supplementary Table 2.a. Median effective dose coefficients (in mSv/mGy-cm) for combined patient age and patient diameter strata. Head scans.

| Diameter (cm) | | | | | |
| --- | --- | --- | --- | --- | --- |
| Patient Age | 10-11 cm | 12-13 cm | 14-15 cm | 16-17 cm | 18-19 cm |
| 0 Yrs | 0.038 | 0.041 | 0.042 | 0.033 | 0.034 |
| 1 Yrs | 0.011 | 0.015 | 0.017 | 0.020 |  |
| 2 Yrs | 0.012 | 0.014 | 0.018 | 0.018 |  |
| 3 Yrs | 0.007 | 0.008 | 0.011 | 0.011 |  |
| 4 Yrs | 0.007 | 0.008 | 0.011 | 0.012 | 0.013 |
| 5 Yrs | 0.007 | 0.008 | 0.011 | 0.012 | 0.012 |
| 6 Yrs | 0.007 | 0.008 | 0.008 | 0.011 | 0.014 |
| 7 Yrs | 0.007 | 0.007 | 0.007 | 0.008 | 0.018 |
| 8 Yrs | 0.004 | 0.005 | 0.005 | 0.005 | 0.009 |
| 9 Yrs | 0.004 | 0.005 | 0.005 | 0.005 | 0.007 |
| 10 Yrs | 0.004 | 0.005 | 0.004 | 0.005 | 0.006 |
| 11 Yrs | 0.004 | 0.005 | 0.005 | 0.005 | 0.006 |
| 12 Yrs | 0.003 | 0.004 | 0.005 | 0.005 | 0.006 |
| 13 Yrs | 0.003 | 0.003 | 0.003 | 0.003 | 0.004 |
| 14 Yrs | 0.003 | 0.003 | 0.003 | 0.003 | 0.004 |
| 15 Yrs | 0.003 | 0.003 | 0.003 | 0.003 | 0.004 |
| 16 Yrs | 0.002 | 0.003 | 0.003 | 0.003 | 0.004 |
| 17 Yrs | 0.003 | 0.003 | 0.003 | 0.003 | 0.004 |
| 18 Yrs |  | 0.004 | 0.003 | 0.003 | 0.004 |
| 19 Yrs |  | 0.003 | 0.003 | 0.003 | 0.003 |
| 20 Yrs |  | 0.003 | 0.003 | 0.003 | 0.004 |
| 21 Yrs |  |  | 0.003 | 0.003 | 0.003 |

Supplementary Table 2.b. Median effective dose coefficients (in mSv/mGy-cm) for combined patient age and patient diameter strata. Neck scans.

| Diameter (cm) | | | | | | | |
| --- | --- | --- | --- | --- | --- | --- | --- |
| Patient Age | 10-11 cm | 12-13 cm | 14-15 cm | 16-17 cm | 18-19 cm | 20-21 cm | 22-23 cm |
| 0 Yrs | 0.162 | 0.207 |  |  |  |  |  |
| 1 Yrs | 0.143 | 0.189 | 0.190 |  |  |  |  |
| 2 Yrs | 0.133 | 0.188 | 0.203 |  |  |  |  |
| 3 Yrs | 0.114 | 0.146 | 0.196 |  |  |  |  |
| 4 Yrs | 0.114 | 0.105 | 0.135 |  |  |  |  |
| 5 Yrs | 0.112 | 0.149 | 0.164 | 0.089 |  |  |  |
| 6 Yrs | 0.076 | 0.085 | 0.136 | 0.175 |  |  |  |
| 7 Yrs | 0.078 | 0.071 | 0.086 | 0.079 | 0.168 |  |  |
| 8 Yrs | 0.070 | 0.066 | 0.079 | 0.093 | 0.080 | 0.096 |  |
| 9 Yrs | 0.064 | 0.062 | 0.069 | 0.078 | 0.080 |  |  |
| 10 Yrs | 0.058 | 0.057 | 0.065 | 0.073 | 0.064 | 0.058 |  |
| 11 Yrs | 0.048 | 0.049 | 0.056 | 0.065 | 0.057 | 0.059 |  |
| 12 Yrs | 0.036 | 0.034 | 0.042 | 0.041 | 0.042 | 0.035 |  |
| 13 Yrs | 0.024 | 0.027 | 0.032 | 0.032 | 0.035 | 0.035 | 0.025 |
| 14 Yrs | 0.021 | 0.021 | 0.026 | 0.030 | 0.033 | 0.030 | 0.028 |
| 15 Yrs | 0.019 | 0.020 | 0.027 | 0.029 | 0.030 | 0.029 | 0.029 |
| 16 Yrs | 0.018 | 0.019 | 0.026 | 0.028 | 0.026 | 0.031 | 0.030 |
| 17 Yrs | 0.019 | 0.019 | 0.026 | 0.024 | 0.024 | 0.028 | 0.031 |
| 18 Yrs | 0.024 | 0.024 | 0.020 | 0.027 | 0.022 | 0.032 |  |
| 19 Yrs | 0.019 | 0.020 | 0.024 | 0.025 | 0.027 |  |  |
| 20 Yrs | 0.030 | 0.032 | 0.032 | 0.036 |  |  |  |
| 21 Yrs | 0.038 | 0.028 | 0.025 | 0.030 |  |  |  |

Supplementary Table 2.c. Median effective dose coefficients (in mSv/mGy-cm) for combined patient age and patient diameter strata. Chest scans.

| Diameter (cm) | | | | | | | | | | | | | |
| --- | --- | --- | --- | --- | --- | --- | --- | --- | --- | --- | --- | --- | --- |
| Patient Age | 10-11 cm | 12-13 cm | 14-15 cm | 16-17 cm | 18-19 cm | 20-21 cm | 22-23 cm | 24-25 cm | 26-27 cm | 28-29 cm | 30-31 cm | 32-33 cm | 34-35 cm |
| 0 Yrs | 0.325 | 0.287 | 0.176 |  |  |  |  |  |  | 0.093 |  |  |  |
| 1 Yrs |  | 0.180 | 0.164 | 0.170 |  |  |  |  |  |  |  |  |  |
| 2 Yrs |  | 0.285 | 0.162 | 0.151 |  |  |  |  |  |  |  |  |  |
| 3 Yrs |  | 0.160 | 0.145 | 0.135 | 0.105 |  |  |  |  |  |  |  |  |
| 4 Yrs |  |  | 0.138 | 0.128 | 0.124 |  |  |  |  |  |  |  |  |
| 5 Yrs |  |  | 0.139 | 0.129 | 0.125 | 0.083 |  |  |  |  |  |  |  |
| 6 Yrs |  |  | 0.131 | 0.120 | 0.118 | 0.091 | 0.069 |  |  |  |  |  |  |
| 7 Yrs |  |  | 0.136 | 0.107 | 0.105 | 0.092 | 0.082 |  |  |  |  |  |  |
| 8 Yrs |  |  |  | 0.108 | 0.099 | 0.087 | 0.079 |  |  |  |  |  |  |
| 9 Yrs |  |  |  | 0.092 | 0.091 | 0.088 | 0.078 | 0.069 | 0.056 |  |  |  |  |
| 10 Yrs |  |  |  | 0.088 | 0.092 | 0.083 | 0.076 | 0.054 | 0.058 |  |  |  |  |
| 11 Yrs |  |  |  | 0.085 | 0.085 | 0.076 | 0.070 | 0.058 | 0.048 | 0.053 |  |  |  |
| 12 Yrs |  |  |  |  | 0.075 | 0.069 | 0.065 | 0.051 | 0.051 | 0.049 | 0.034 |  |  |
| 13 Yrs |  |  |  |  | 0.074 | 0.068 | 0.059 | 0.050 | 0.047 | 0.042 | 0.041 |  |  |
| 14 Yrs |  |  |  | 0.061 | 0.056 | 0.061 | 0.052 | 0.046 | 0.042 | 0.038 | 0.035 | 0.031 | 0.027 |
| 15 Yrs |  |  |  | 0.050 | 0.056 | 0.059 | 0.053 | 0.045 | 0.041 | 0.037 | 0.035 | 0.031 |  |
| 16 Yrs |  |  |  |  |  | 0.057 | 0.051 | 0.046 | 0.040 | 0.038 | 0.036 | 0.030 | 0.029 |
| 17 Yrs |  |  |  |  | 0.051 | 0.049 | 0.049 | 0.044 | 0.040 | 0.037 | 0.034 | 0.031 | 0.023 |
| 18 Yrs |  |  |  |  |  |  |  | 0.039 | 0.032 | 0.025 |  |  |  |
| 19 Yrs |  |  |  |  |  |  |  | 0.045 | 0.039 | 0.036 | 0.038 |  |  |
| 20 Yrs |  |  |  |  |  |  | 0.053 | 0.049 | 0.044 | 0.046 | 0.042 |  |  |
| 21 Yrs |  |  |  |  |  |  |  | 0.045 | 0.038 | 0.035 | 0.033 |  |  |

Supplementary Table 2.d. Median effective dose coefficients (in mSv/mGy-cm) for combined patient age and patient diameter strata. Cardiac scans.

| Diameter (cm) | | | | |
| --- | --- | --- | --- | --- |
| Patient Age | 10-11 cm | 12-13 cm | 14-15 cm | 16-17 cm |
| 0 Yrs | 0.377 | 0.353 | 0.343 |  |
| 1 Yrs | 0.345 | 0.343 | 0.304 |  |
| 2 Yrs |  |  | 0.309 |  |
| 3 Yrs |  | 0.291 |  | 0.255 |

Supplementary Table 2.e. Median effective dose coefficients (in mSv/mGy-cm) for combined patient age and patient diameter strata. Abdomen and pelvis scans.

| Diameter (cm) | | | | | | | | | | | | | |
| --- | --- | --- | --- | --- | --- | --- | --- | --- | --- | --- | --- | --- | --- |
| Patient Age | 12-13 cm | 14-15 cm | 16-17 cm | 18-19 cm | 20-21 cm | 22-23 cm | 24-25 cm | 26-27 cm | 28-29 cm | 30-31 cm | 32-33 cm | 34-35 cm | 36-37 cm |
| 0 Yrs | 0.085 | 0.090 | 0.094 | 0.063 |  |  | 0.070 |  |  |  |  |  |  |
| 1 Yrs | 0.095 | 0.099 | 0.092 |  |  |  |  |  |  |  |  |  |  |
| 2 Yrs | 0.091 | 0.095 | 0.093 | 0.085 |  |  |  |  |  |  |  |  |  |
| 3 Yrs | 0.097 | 0.088 | 0.085 | 0.092 |  |  |  |  |  |  |  |  |  |
| 4 Yrs | 0.085 | 0.085 | 0.081 | 0.071 | 0.063 |  |  |  |  |  |  |  |  |
| 5 Yrs |  | 0.082 | 0.078 | 0.070 | 0.061 | 0.056 |  |  |  |  |  |  |  |
| 6 Yrs |  | 0.067 | 0.063 | 0.061 | 0.058 | 0.049 | 0.048 |  |  |  |  |  |  |
| 7 Yrs |  | 0.072 | 0.059 | 0.056 | 0.048 | 0.047 | 0.033 | 0.041 |  |  |  |  |  |
| 8 Yrs |  | 0.062 | 0.062 | 0.054 | 0.050 | 0.044 | 0.042 | 0.044 | 0.035 |  |  |  |  |
| 9 Yrs |  | 0.046 | 0.053 | 0.051 | 0.045 | 0.040 | 0.036 | 0.034 | 0.035 | 0.029 |  |  |  |
| 10 Yrs |  |  | 0.050 | 0.051 | 0.045 | 0.038 | 0.037 | 0.032 | 0.026 | 0.028 |  |  |  |
| 11 Yrs |  |  | 0.044 | 0.044 | 0.040 | 0.034 | 0.031 | 0.030 | 0.027 | 0.026 | 0.024 | 0.021 |  |
| 12 Yrs |  |  | 0.034 | 0.038 | 0.036 | 0.032 | 0.027 | 0.027 | 0.024 | 0.022 | 0.020 | 0.022 |  |
| 13 Yrs |  |  | 0.041 | 0.036 | 0.033 | 0.030 | 0.027 | 0.026 | 0.022 | 0.020 | 0.022 | 0.016 |  |
| 14 Yrs |  |  | 0.031 | 0.033 | 0.033 | 0.029 | 0.026 | 0.024 | 0.022 | 0.021 | 0.017 | 0.014 | 0.014 |
| 15 Yrs |  |  | 0.031 | 0.031 | 0.033 | 0.029 | 0.025 | 0.023 | 0.020 | 0.019 | 0.019 | 0.016 | 0.019 |
| 16 Yrs |  |  | 0.021 | 0.028 | 0.031 | 0.027 | 0.024 | 0.023 | 0.022 | 0.018 | 0.017 | 0.016 | 0.017 |
| 17 Yrs |  |  |  | 0.030 | 0.028 | 0.027 | 0.024 | 0.020 | 0.020 | 0.016 | 0.015 | 0.015 | 0.019 |
| 18 Yrs |  |  |  |  |  | 0.025 | 0.022 | 0.020 | 0.022 | 0.016 | 0.018 | 0.020 | 0.012 |
| 19 Yrs |  |  |  |  |  | 0.027 | 0.020 | 0.019 | 0.018 | 0.017 | 0.019 |  |  |
| 20 Yrs |  |  |  |  | 0.032 | 0.031 | 0.031 | 0.026 | 0.024 | 0.021 | 0.019 | 0.020 | 0.017 |
| 21 Yrs |  |  |  |  | 0.028 | 0.029 | 0.029 | 0.026 | 0.024 | 0.020 | 0.020 | 0.017 | 0.017 |

Supplementary Table 2.f. Median effective dose coefficients (in mSv/mGy-cm) for combined patient age and patient diameter strata. Combined chest abdomen and pelvis scans.

| Diameter (cm) | | | | | | | | | | | | |
| --- | --- | --- | --- | --- | --- | --- | --- | --- | --- | --- | --- | --- |
| Patient Age | 12-13 cm | 14-15 cm | 16-17 cm | 18-19 cm | 20-21 cm | 22-23 cm | 24-25 cm | 26-27 cm | 28-29 cm | 30-31 cm | 32-33 cm | 34-35 cm |
| 0 Yrs | 0.133 | 0.175 |  |  |  |  |  |  |  |  |  |  |
| 1 Yrs | 0.121 | 0.103 | 0.101 |  |  |  |  |  |  |  |  |  |
| 2 Yrs | 0.096 | 0.097 | 0.090 |  |  |  |  |  |  |  |  |  |
| 3 Yrs | 0.130 | 0.090 | 0.092 | 0.073 |  |  |  |  |  |  |  |  |
| 4 Yrs |  | 0.080 | 0.079 | 0.072 |  |  |  |  |  |  |  |  |
| 5 Yrs |  | 0.079 | 0.077 | 0.067 |  |  |  |  |  |  |  |  |
| 6 Yrs |  | 0.082 | 0.071 | 0.064 | 0.044 |  |  |  |  |  |  |  |
| 7 Yrs |  | 0.096 | 0.071 | 0.065 | 0.053 | 0.052 |  |  |  |  |  |  |
| 8 Yrs |  | 0.145 | 0.071 | 0.064 | 0.058 |  |  |  |  |  |  |  |
| 9 Yrs |  |  | 0.064 | 0.058 | 0.054 |  |  |  |  |  |  |  |
| 10 Yrs |  | 0.061 | 0.065 | 0.053 | 0.049 | 0.039 | 0.051 |  |  |  |  |  |
| 11 Yrs |  | 0.055 |  | 0.054 | 0.050 | 0.041 |  |  |  |  |  |  |
| 12 Yrs |  | 0.053 | 0.046 | 0.053 | 0.043 | 0.040 | 0.034 | 0.030 | 0.030 | 0.027 |  |  |
| 13 Yrs | 0.043 | 0.044 | 0.039 | 0.049 | 0.043 | 0.035 | 0.033 | 0.029 | 0.025 |  |  |  |
| 14 Yrs | 0.039 | 0.036 | 0.041 | 0.045 | 0.037 | 0.035 | 0.031 | 0.027 | 0.024 | 0.021 |  |  |
| 15 Yrs |  | 0.036 | 0.042 | 0.038 | 0.040 | 0.034 | 0.030 | 0.029 | 0.024 | 0.023 | 0.020 |  |
| 16 Yrs |  | 0.034 | 0.042 | 0.032 | 0.036 | 0.035 | 0.031 | 0.027 | 0.025 | 0.022 | 0.020 | 0.019 |
| 17 Yrs | 0.041 | 0.035 | 0.034 | 0.032 | 0.035 | 0.035 | 0.031 | 0.028 | 0.027 | 0.024 | 0.020 | 0.021 |
| 18 Yrs |  |  |  |  |  | 0.034 | 0.032 |  |  |  |  |  |
| 19 Yrs |  |  |  |  |  |  | 0.032 | 0.028 | 0.025 |  |  |  |
| 20 Yrs |  |  |  |  |  |  | 0.033 | 0.024 | 0.021 |  |  |  |
| 21 Yrs |  |  |  |  |  |  | 0.026 | 0.022 | 0.024 | 0.019 | 0.017 |  |

Supplementary Table 2.g. Median effective dose coefficients (in mSv/mGy-cm) for combined patient age and patient diameter strata. Thoracic or lumbar spine scans.

| Diameter (cm) | | | | | | | | |
| --- | --- | --- | --- | --- | --- | --- | --- | --- |
| Patient Age | 10-11 cm | 12-13 cm | 14-15 cm | 16-17 cm | 18-19 cm | 20-21 cm | 22-23 cm | 24-25 cm |
| 4 Yrs | 0.061 |  |  |  |  |  |  |  |
| 5 Yrs | 0.068 |  |  |  |  |  |  |  |
| 7 Yrs |  | 0.047 |  |  |  |  |  |  |
| 8 Yrs | 0.055 | 0.039 |  |  |  |  |  |  |
| 9 Yrs |  | 0.036 | 0.042 |  |  |  |  |  |
| 10 Yrs | 0.048 | 0.047 | 0.040 |  |  |  |  |  |
| 11 Yrs | 0.042 | 0.043 | 0.038 | 0.034 |  |  |  |  |
| 12 Yrs | 0.036 | 0.039 | 0.036 | 0.027 | 0.023 |  |  |  |
| 13 Yrs | 0.035 | 0.039 | 0.032 | 0.027 | 0.034 | 0.026 |  | 0.023 |
| 14 Yrs | 0.029 | 0.025 | 0.024 | 0.025 | 0.026 | 0.024 | 0.030 |  |
| 15 Yrs | 0.031 | 0.028 | 0.023 | 0.025 | 0.027 | 0.024 | 0.024 | 0.021 |
| 16 Yrs | 0.029 | 0.023 | 0.020 | 0.021 | 0.022 | 0.021 | 0.023 | 0.022 |
| 17 Yrs | 0.021 | 0.022 | 0.022 | 0.022 | 0.021 | 0.023 | 0.024 | 0.029 |
| 19 Yrs |  |  | 0.028 |  |  |  |  |  |
| 21 Yrs |  |  |  |  | 0.028 |  |  |  |
